# Lectin-Binding Specificity of the Fertilization-Relevant Protein PDC-109 by Means of Surface Plasmon Resonance and Carbohydrate REcognition Domain EXcision-Mass Spectrometry

**DOI:** 10.3390/ijms19041076

**Published:** 2018-04-04

**Authors:** Sira Defaus, Manuel Avilés, David Andreu, Ricardo Gutiérrez-Gallego

**Affiliations:** 1 Department of Experimental and Health Sciences, Pompeu Fabra University, Barcelona Biomedical Research Park, 08003 Barcelona, Spain; sira.defaus@upf.edu; 2 Department of Cell Biology and Histology, School of Medicine, University of Murcia and IMIB-Arrixaca, Campus Mare Nostrum, 30071 Murcia, Spain; maviles@um.es

**Keywords:** PDC-109, carbohydrate, SPR, CREDEX-MS, lectin-binding profile

## Abstract

Seminal plasma proteins are relevant for sperm functionality and some appear responsible for establishing sperm interactions with the various environments along the female genital tract towards the oocyte. In recent years, research has focused on characterizing the role of these proteins in the context of reproductive biology, fertility diagnostics and treatment of related problems. Herein, we focus on the main protein of bovine seminal plasma, PDC-109 (BSP-A1/-A2), which by virtue of its lectin properties is involved in fertilization. By means of surface plasmon resonance, the interaction of PDC-109 with a panel of the most relevant glycosidic epitopes of mammals has been qualitatively and quantitatively characterized, and a higher affinity for carbohydrates containing fucose has been observed, in line with previous studies. Additionally, using the orthogonal technique of Carbohydrate REcognition Domain EXcision-Mass Spectrometry (CREDEX-MS), the recognition domain of the interaction complexes between PDC-109 and all fucosylated disaccharides [(Fuc-α1,(3,4,6)-GlcNAc)] has been defined, revealing the specific glycotope and the peptide domain likely to act as the PDC-109 carbohydrate binding site.

## 1. Introduction

In the male genital tract, secretions from the testes, epididymis, seminal vesicles, and other accessory glands contribute to the fluid portion of semen (i.e., seminal plasma), in which sperm cells (i.e., spermatozoa) are suspended. Mammal seminal plasma is a complex fluid that serves as carrier for the spermatozoa in their journey to their target, the uterus. However, since the beginning of the 20th century, researchers realized that seminal plasma played a more important role in sperm biology than that of a mere vehicle for spermatozoa. Growing evidence has pointed to this “acellular” part of the semen as a fundamental player in mammalian fertilization, and in recent years intensive research has led to the identification and functional characterization of several seminal plasma proteins. Since sperm cells have a limited biosynthetic ability [1], the interaction of different seminal plasma proteins with spermatozoa, as well as with proteins from the female genital tract encountered during the journey to the site of fertilization, will have a profound impact on sperm functionality. Proteins from the seminal fluid will be adsorbed on the sperm surface, remodeling the structure of sperm membrane protein domains. These changes will endow spermatozoa not only with the ability to fertilize the egg but will also influence several essential steps of the fertilization process, such as sperm capacitation, establishment of an oviductal sperm reservoir, modulation of the uterine immune environment and, ultimately, gamete interaction [2]. Therefore, studies on the nature, structure, and functional properties of seminal plasma protein constituents, and on their interactions with molecular ligands and spermatozoa, might provide clues on how seminal plasma modulates the functional ability of spermatozoa. These insights are likely to help advance aspects of such as diagnostics and treatment of reproductive dysfunction, as semen ultimately reflects the status of the male reproductive organs.

The bovine seminal plasma proteome is highly complex [3] but has a major fraction consisting of four acidic bovine seminal plasma (BSP) proteins designated BSP-Al, BSP-A2, BSP-A3, and BSP-30 kDa [4], secreted by the seminal vesicles and characterized by a common conserved fibronectin type II domain. Similar proteins have been found in seminal plasma and/or seminal vesical secretions from humans [5], hamsters, mice, rats [6], stallions [7], and boars [8], suggesting that BSP proteins are ubiquitous in mammals and may possibly be involved in a common function. These proteins constitute the major heparin-binding protein fraction of fresh bovine seminal fluid (30–50 mg/mL), and together comprise an average 47% of the total protein fraction [9]. In particular, BSP-A1 and BSP-A2 are present in roughly equimolar concentrations, have identical amino acid sequence and differ only in the degree of glycosylation (with or without an O-linked trisaccharide); thus, they have been classified as a single chemical entity named PDC-109, representing on average a 38% of the total protein content and the most abundant heparin-binding BSP proteins [10]. At ejaculation, around 8 million PDC-109 molecules bind to the sperm surface, mainly to choline phospholipids [11] that comprise over 70% of total BSP membrane phospholipids. In view of its high abundance in BSP and its binding to spermatozoa, PDC-109 has been investigated extensively by various biochemical and biophysical approaches that have outlined its binding properties and sperm capacitation potential [12,13]. Also, strong evidence supports the involvement of PDC-109 in sperm reservoir formation, acting as a lectin that recognizes Fuc residues in the epithelium. In fact, a Fuc-binding molecule purified from sperm extracts and identified as PDC-109 [14,15] was shown to enable sperm attachment to the oviductal epithelium in a complex quaternary binding event. 

The purpose of the present study is to further unravel the lectin-binding profile of PDC-109 by evaluating its specificity against hitherto unreported glycan epitopes, as well as to define the carbohydrate recognition domain (CRD) of PDC-109 to gain insights into its role along the fertilization process.

## 2. Results

### 2.1. Lectin Profile of PDC-109 by Surface Plasmon Resonance (SPR)

#### 2.1.1. Synthesis of Neo-Glycoprobes Exposing Relevant Mammalian Carbohydrate Epitopes

We aimed to study the lectin-binding profile of PDC-109 using purified protein material from biological origin, hence initial work was focused on the synthesis, purification and validation of new glycoprobes displaying some of the most relevant glycotopes in the mammalian system (Table 1).

All the glycopeptides were synthesized as previously reported by our group [16] using oxime ligation between the oligosaccharides in Table 1 and the peptide module *N*[Me]-*O*-Aoa-GFKKG at 25 and 20 mM, respectively. Ligation reactions were performed at pH 3.5 (NAc-hexoses) or pH 4.6 (hexoses) and 37 °C for 72 h, the conjugates purified by high performance liquid chromatography (HPLC) and characterized by mass spectrometry (MS) as detailed in Materials and Methods. In addition to choosing an optimal pH for every hexose type, the use of aniline as catalyst to reduce incubation time was evaluated, as some authors have reported significant yield improvements (nearly double in 3 h) in conjugations involving non-methylated aminoxyacetic acid (Aoa)-containing peptides and monosaccharides [17]. However, with our *N*[Me]-*O*-Aoa-peptide the yield increased only marginally after 72 h incubation, except for reactions with Fuc-containing i.e., [Fuc-α1,(3,4,6)-GlcNAc] disaccharides (e.g., from 8% to 16% for Fuc-α1,4-GlcNAc).

A substantial problem during the production of the glycopeptides was the purification by reversed phase (RP) HPLC, as the small change in hydrophobicity between the glycopeptide product and unreacted *N*[Me]-*O*-Aoa-peptide precursor rendered separations difficult, particularly in conjugations with <20% conversion. Attempts to increase the retention time of unreacted peptide by addition of carbonyl scavengers such as formaldehyde and acetone, or the alkylating agent *N*-ethylmaleimide [18], did not improve resolution. An additional difficulty, also related to purification, was that residual (trifluoroacetic) acid from HPLC eluents caused degradation of the *N*[Me]-*O*-Aoa-peptides during lyophilization. This problem was conveniently solved by adjusting to pH 5 the glycoprobe solution immediately after collection. A further test of the suitability of the conjugation conditions involved the highly acid-labile trisaccharides containing terminal sialic (*N*-acetyl neuraminic) acid (Sia), which could be successfully conjugated to the *N*[Me]-*O*-Aoa-peptide module at mildly acidic pH, purified and characterized by MS like other glycoconjugates. Finally, the long-term stability of all compounds was shown to be satisfactory (>95% unaltered after 2 years) provided they are stored in lyophilized form. This renders our glycopeptide probe approach useful for longitudinal studies in carbohydrate research.

#### 2.1.2. Neo-Glycoprobe Evaluation by SPR Studies with Known Lectins

With neo-glycopeptides displaying the most relevant glycotopes of the mammalian system in hand, their functionality was validated in SPR interaction studies using plant lectins (Table 1) with known selectivity for the indicated carbohydrate epitopes. Lectin solutions were flown across a surface with the immobilized cognate sugar unit. Ac-GFKKG-amide, a non-glycosylated probe, was used as reference surface, given that the previously described *N*[Me]-*O*-Aoa-GFKKG-amide [16] displayed undesirable, non-specific binding with PDC-109.

Disaccharides with terminal β-galactose were first evaluated. As in our proof of concept publication [16], a sensor chip with the same β-galactosides [i.e., Gal(β1-4)GlcNAc, Gal(β1-3)GlcNAc, Gal(β1-6)GlcNAc)] and the new Ac-GFKKG-amide reference surface was prepared. Interaction between β-galactose-specific lectin ECA and these β-galactosides yielded the same kinetic results [16], hence confirmed sensor surface functionality.

**Figure 1:**
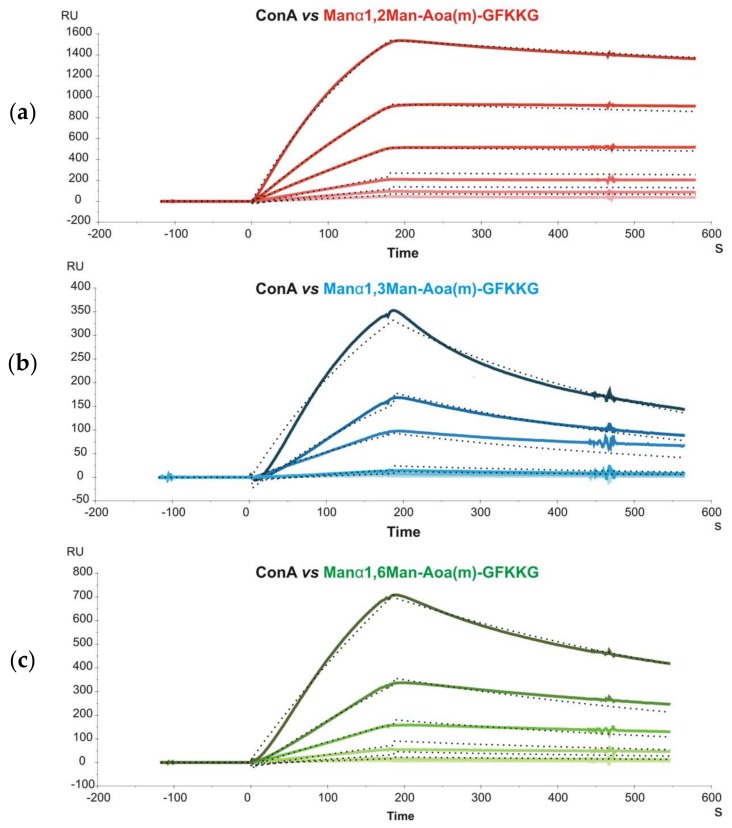
Binding of Con A to immobilized mannobiose-containing glycoprobes ((**a**) Man-α1,2-Man; (**b**) Man-α1,3-Man; (**c**) Man-α1,6-Man) at six different concentrations (2.5, 5, 10, 20, 40 and 80 nM). Fitting curves using a bivalent model are shown as dotted black lines.

Subsequently, glycoprobes displaying mannobioses only differing in their glycosidic linkage (Man-α1,2-Man, Man-α1,3-Man and Man-α1,6-Man) were immobilized on a sensor chip and their interaction with *concanavalin A* (Con A) was studied. Con A is a relatively complex lectin, organized as a β-barrel-like tetramer at pH > 7, each dimer subunit (D2 symmetry) with two CRDs situated on opposing faces of the protein. Sensorgrams of different Con A concentrations were recorded and kinetic constants determined by simultaneously fitting the experimental curves to a bivalent kinetic model (Figure 1). This model allows the first and second binding events to be separately described by two sets of rate constants (Table 2).

The unconventional response units (RU^−1^) used to describe the second association event made comparison with constants derived from other methods (e.g., Isothermal Titration Calorimetry, ITC) difficult, and only the affinity constants for the first event, K_A1_ (K_A1_ = k_a1_/k_d1_) could be accurately compared. For this event, a higher affinity of Man-α1,3-Man over Man-α1,6-Man was found, in agreement with previous ITC data [19], although the K_A1_ values obtained with the ITC method were one order of magnitude lower (~10^4^) than with our SPR approach (~10^5^). One possible explanation for this difference, keeping in mind the two-CRDs-per-dimer model of Con A, is that although the bivalent binding model used in SPR only allows to derive standard-unit values for K_A1_, the apparent k_d1_, used for K_A1_ determination is also influenced by the second event. If this contribution is not factored in, a lower k_d1_, and consequently a higher K_A1_, results.

**Figure 2:**
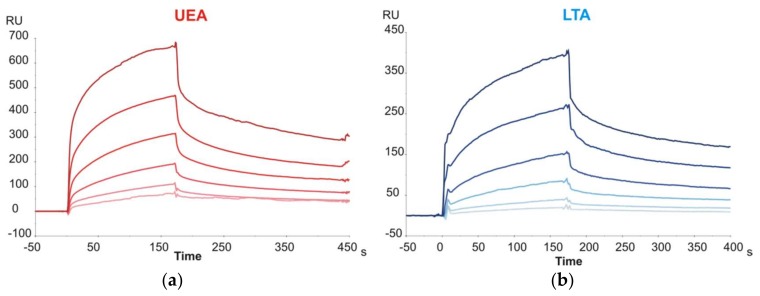
Binding sensorgrams of two Fuc-binding lectins ((**a**) UEA; (**b**) LTA) at six different concentrations from 31.25 nM to 1 µM, to Fuc-α1,4-GlcNAc–glycoprobe.

The last group of disaccharide glycoprobes to be evaluated included those with terminal Fuc units (Fuc-α1,3-GlcNAc, Fuc-α1,4-GlcNAc and Fuc-α1,6-GlcNAc). The latter epitope corresponds to the core-fucosylation of N-glycans, and the former two are partial Lewis^x^ and Lewis^a^ epitopes, respectively. The glycoprobe surfaces were tested against two Fuc-specific lectins from *Lotus tetragonolobus* (LTA) and *Ulex europeaus* (UEA). For both lectins, sensorgrams clearly demonstrated affinity for Fuc epitopes (Figure 2; representative example for Fuc-α1,4-GlcNAc), even though the carbohydrate affinity for both lectins has been described to be strongly specific for the blood group H determinant (Fuc-α1,2-Gal) [20,21], not considered in our study.

**Figure 3:**
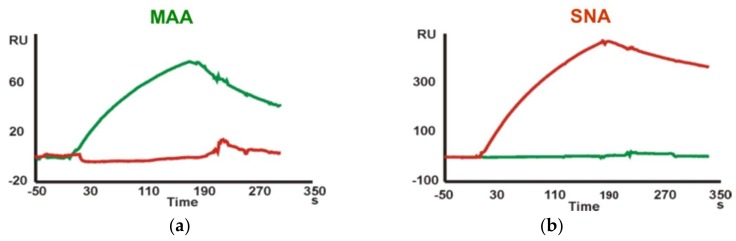
Binding sensorgrams showing the different carbohydrate specificity of both lectins ((**a**) MAA; (**b**) SNA) to their specific sugar epitopes (Neu5Ac-α2,3-lacNAc and Neu5Ac-α2,6-lacNAc in green and red trace, respectively).

Finally, kinetic data for the interaction of Neu5Ac-containing trisaccharide glycoprobes with the *Maackia amurensis* (MAA) and *Sambucus nigra* (SNA) lectins were determined. A sensor chip with the Neu5Ac-α2,3-Gal-β1,4-GlcNAc-*N*[Me]-*O*-Aoa-GFKKG-amide, Neu5Ac-α2,6-Gal-β1,4-GlcNAc-*N*[Me]-*O*-Aoa-GFKKG-amide and Neu5Ac-α2,3/6-Gal-β1,4-Glc-*N*[Me]-*O*-Aoa-GFKKG-amide glycoprobes, representative of the two existing linkage types in adult mammalian glycoproteins, was prepared and the two Sia-specific lectins were flown over the surfaces. As the MAA lectin reportedly requires three intact sugar units for binding [22], our results support a native ring-closed structure for the first monosaccharide. The binding responses observed for the two lectins were in perfect agreement with their reported carbohydrate specificity. Thus, whereas SNA showed a marked preference for the Sia-α2,6-lacNAc isomer, MAA recognized only the Sia-α2,3-lacNAc containing glycoprobe (Figure 3). 

**Figure 4:**
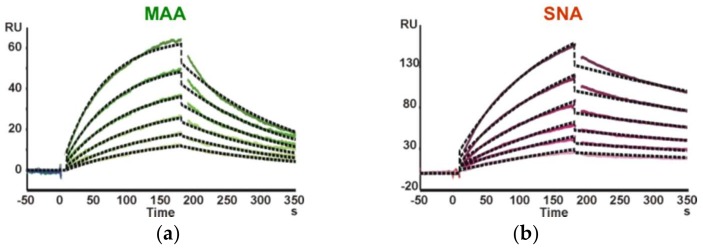
SPR sensorgrams of the two Sia-specific lectins at six different concentrations. (**a**) Binding of MAA to Neu5Ac-α2,3-lacNAc glycoprobe; (**b**) Binding of SNA to Neu5Ac-α2,6-lacNAc glycoprobe. Fitted curves are indicated as dotted black lines.

For kinetic analysis, several concentrations in the 250 nM to 1.9 μM range for MAA and in the 74 to 563 nM range for SNA were analyzed. A 1:1 Langmuir binding model was used for sensorgram fitting (Figure 4, Table 3). As expected for interactions involving trisaccharides, the K_A_ equilibrium constants obtained were in the 10^6^–10^7^ M^−1^ range. For MAA, the affinity constant determined by our approach was 9.12 × 10^5^ M^−1^; of the same magnitude than reported previously [23], but one order lower than other constants determined using complex neoglycoproteins and immobilized lectins [24], in both cases by SPR. The higher affinity for neoglycoproteins can easily be explained by their multivalent nature. For SNA, a K_A_ of 6.27 × 10^6^ M^−1^ was determined, consistent with the previously reported value [25] of 6.7 × 10^6^ M^−1^.

In conclusion, the kinetic and affinity constants of well-known plant lectins for their specific carbohydrate ligands determined by our SPR approach were consistent with previously reported data, corroborating that the glycoprobes attached to sensor surface chips provide a reliable setting for accurate determination of the carbohydrate binding profile of the mammalian lectin PDC-109.

#### 2.1.3. PDC-109 Binding Profile and Kinetic Studies

While the lipid selectivity and kinetics of PDC-109 has been characterized in considerable detail [11,26], its interaction with carbohydrate epitopes remains largely uncharted, despite its potential role in sperm reservoir formation in the female genital tract. We have therefore performed SPR experiments with PDC-109 and the most relevant mammalian glycotopes, particularly those containing Fuc epitopes. Once SPR sensor chips were functionalized with the different synthetic glycoprobes and evaluated with specific lectins (Section 2.1.2) they were used to characterize both qualitatively and quantitatively the interaction of PDC-109. For kinetic analysis, several concentrations in the 6.25 to 100 μM range were analyzed and a 1:1 Langmuir binding model was chosen for sensorgram fitting.

**Figure 5:**
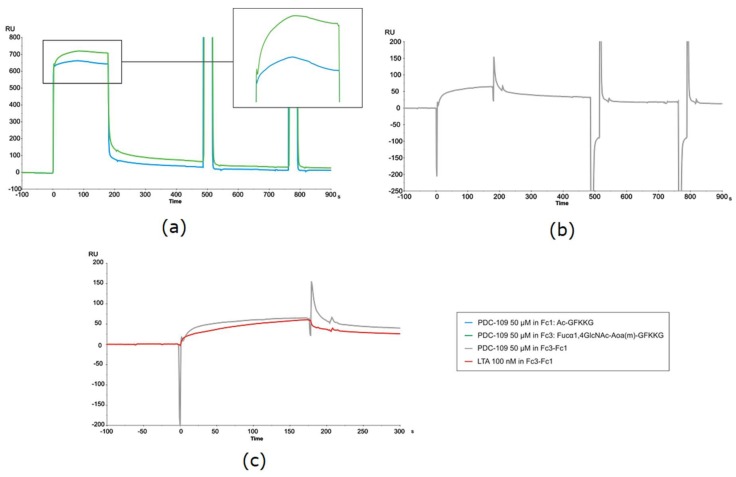
(**a**) SPR sensorgrams of the binding of PDC-109 at 50 μM to reference surface, i.e., Ac-GFKKG (blue trace), or to Fuc-α1,4-GlcNAc-Aoa(m)-GFKKG glycoprobe immobilized (green trace). Enlarged on the right, the unusual signal progression in association phase is highlighted; (**b**) Differential curve for PDC-109 kinetic studies obtained after subtracting the reference channel; (**c**) Comparison of differential curves from PDC-109 (grey trace) vs. lectin LTA (red trace).

In general, considerable variability was observed in the kinetic experiments, with CV (from 3 to 6 repeat experiments) exceeding 50% in particular cases. Data dispersion was attributable to the unusual sensorgrams observed, reflecting a complex binding event, particularly in the association phase of PDC-109. As shown in Figure 5a, the usual signal rise observed right after sample injection and due to bulk effects—refractive index differences between analyte solution and flow buffer—was followed by a rather intriguing association phase. In a conventional, straightforward binding event, the response—i.e., the signal rise over time, reflecting protein binding to the sensor surface—reaches a constant value once equilibrium is established, and remains so until the protein solution passed over the sensor chip is replaced with buffer, giving way to the spontaneous dissociation process and subsequent surface regeneration. However, in the present case visual inspection of all PDC-109 sensorgrams (those in Figure 5a being representative) clearly shows a biphasic phenomenon during the association phase (displayed enlarged in Figure 5a).

Thus, response increased until a high value (maximum binding) was reached, then slowly decreased even though PDC-109 was still being flown across the surface. Such biphasic behavior during the association phase was observed not only with all immobilized glycoprobes but also with the reference surface (Figure 5a; green and blue trace sensorgrams, respectively), hence the differential curve resulting from reference subtraction (Figure 5b) displayed an uneven shape, with large spikes at both the beginning and the end of the injection that complicated data fitting. As a result, values for the kinetic constants may include some variability, although a reliable epitope preference could nonetheless be established. 

This unusual biphasic profile, reflecting loss of mass from the sensor surface during the association phase, was not observed with any of the standard lectins studied earlier (Figure 5c), and suggested an uncharacteristic binding behavior of PDC-109 that could be confirmed after meticulous evaluation of all possible variables (surface, ligand, analyte, buffer conditions, competitive reagents, solute stability, etc.) and with some precedents observed in other systems [26].

Plausible explanations for the unusual behavior of PDC-109, accounting for the biphasic shape of the SPR sensorgrams, would assume the existence of two different affinities, namely two competing analyte reactions, in the protein preparation. This can be due either to a single analyte with two different affinities, or to two different analytes, each with its own affinity.

Regarding the former situation, the possibility of non-specific binding between PDC-109 and the SPR surface was investigated, since the carboxymethyled dextran matrix on the sensor surface is structurally not too distant from the glycoprobe ligands, hence a weak interaction with the lectin could not be excluded. To this end, a “saturated” solution of free carboxymethylated dextran (CM) (identical to that present as CM5 surface coating) was added to the analyte solution; if the analyte had any affinity for either dextran or carboxymethylated variant, it would bind preferentially the free polymer over that on the sensor chip, thus reducing non-specific binding and avoiding competing reactions. This modification, however, did not have any effect on the binding curves, which remained biphasic in all cases. In a complementary attempt, a C1 chip lacking the dextran matrix (only carboxymethyl groups directly bound to the gold surface) was tested to exclude any influence of the matrix; again, the same biphasic profile could be observed during the association phase. From these results, we concluded that no competing reactions with the SPR surface existed, hence the biphasic behavior must proceed from analyte heterogeneity, i.e., two analytes competing for the same ligand. If such was the case, the decreasing region in the association phase curve could result from a smaller molecule with higher ligand affinity but slower kinetics competing with a faster but lower-affinity interaction.

**Figure 6:**
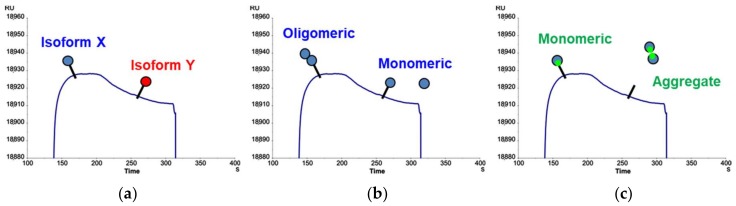
Possible explanations for the biphasic behaviour of PDC-109 due to analyte heterogeneity: (**a**) Different isoforms; (**b**) Different aggregation states; (**c**) Competition from the same binding domain site for both lectin-ligand interaction and formation of protein aggregates. Ligand immobilized on the SPR surface is represented by a stick and analyte (PDC-109) as a circle.

Different possibilities of analyte heterogeneity can be envisaged: (i) PDC-109 may undergo conformational changes upon binding to the epitope; (ii) more than one isoform/glycoform is present in the PDC-109 sample; or (iii) PDC-109 has different aggregation states; all these situations might produce sensorgrams with biphasic association curves, such as those in Figure 6. Previous reports suggest PDC-109 undergoes a conformational change upon binding to PC membranes [27]. However, as SPR can only detect refractive index variations related to mass changes over time, subtle conformational changes not resulting in mass changes will go unnoticed. However, while beyond the scope of the SPR technique, heterogeneity due to conformational change would not explain the SPR signal reduction.

To investigate the possibility of a heterogeneous analyte, several different approaches were followed. Firstly, considering that PDC-109 exists in two major forms (i.e., glycosylated and non-glycosylated) [10], enzymatic deglycosylation was performed in order to obtain a single analyte isoform. Given the limited availability of purified PDC-109, the amount of protein used for deglycosylation did not allow a quantitative purification step. The extent of the reaction was assessed by matrix assisted laser desorption ionization—time of flight (MALDI-TOF) MS, which showed that glycan removal could not be brought to completion. Even so, the partially deglycosylated PDC-109 was tested on the same sensor surfaces previously used with native protein. The resulting sensorgrams clearly showed a rising association phase without biphasic behavior, yet the incomplete deglycosylation prevented formulating clear conclusions, as the standard binding response could not be unequivocally attributed to a single isoform. Secondly, to investigate the possibility of aggregation, PDC-109 was flown across a chip where itself was immobilized. The resulting sensorgrams displayed the previously observed biphasic shape, suggesting that interaction of PDC-109 with itself is the cause of the anomalous biphasic sensorgrams, and that the glycosylated version plays a significant role in this self-recognition, possibly by virtue of the single sialic acid residue present.

Evidence from the literature points out that PDC-109 is naturally produced as a mixture of several protein forms [28] and aggregation states [29] that play an important role in modulating its interaction with other biomolecules. This would allow concluding that, whichever of the situations proposed in Figure 6 applies –including a combination thereof–, PDC-109 acts as a lectin-like molecule albeit with quite unique features that, though not abolishing its ability to recognize carbohydrates, give rise to unusual SPR sensorgrams.

Despite the above-discussed limitations, we were able to determine the SPR binding parameters of PDC-109. Affinity constants (Table 4) indicated weak to moderate binding for carbohydrates, at least if compared to the considerably higher affinity (K_A_~10^7^ M^−1^) found for PC membranes [26]. This would suggest that PDC-109 binding to carbohydrates in oviductal epithelial cells must likely involve some sort of multivalency in order to efficiently retain the sperm in the oviductal reservoir.

From the above data it can also be seen that PDC-109 appears to have the highest binding affinity for Fuc-α1,4-GlcNAc among all glycotopes in this study. This is in agreement with earlier findings which propose that bull sperm binds to an oligosaccharide ligand on the oviductal epithelium that resembles Lewis^a^ trisaccharide (Galβ1-3[Fucα1-4]GlcNAc) [30]. For PDC-109 vs. Fuc-α1,4-GlcNAc, the higher affinity was a consequence of a lower dissociation rate constant, consistent with a privileged interaction with this glycosidic configuration. The observation that PDC-109 displays favored interaction with the Neu5Ac-α2,6-Gal-β1,4-GlcNAc epitope may account for the observed self-recognition. Part of PDC-109 bears an O-linked trisaccharide consistent with this mass [10] and, even though this aspect has never been fully elucidated, it could well be that the Sia residue, when exposed in an α-2,6-configuration, facilitates such binding. Finally, the absence of significant binding with the mannobiose epitopes is in contrast to what was reported by Amari and coworkers. However, they employed oligomannose type pentassacharides and the complex architecture of this oligosaccharide may well account for the difference in binding characteristics. 

### 2.2. Carbohydrate Recognition Domain of PDC-109 by CREDEX-MS

Carbohydrate Recognition Domain Excision (CREDEX) mass spectrometry is a technology to map the interacting domains of sugar-lectin complexes, providing structural information by limited proteolysis (epitope excision/extraction) coupled with MS [31].

In order to test the complementarity of CREDEX-MS with the SPR-based glycoprobe approach for PDC-109, and to define the carbohydrate-binding site of this seminal plasma protein, glycotopes that in the SPR study had displayed higher affinities, i.e., all fucosylated epitopes (Fucα-1,3-GlcNAc, Fucα-1,4-GlcNAc and Fucα-1,6-GlcNAc), were immobilized onto DVS-Sepharose. To guarantee data reliability, prior to the excision experiments with PDC-109, columns were validated for their Fuc-recognition ability by binding experiments with the Fuc-specific UEA-I lectin followed by SDS-PAGE detection. Excision experiments were also carried out and the CRD of UEA-I was successfully delimited (data not shown). The affinity-bound peptides eluted and identified by MS were in agreement with the previously reported structural basis of UEA-I carbohydrate specificity [21].

**Figure 7:**
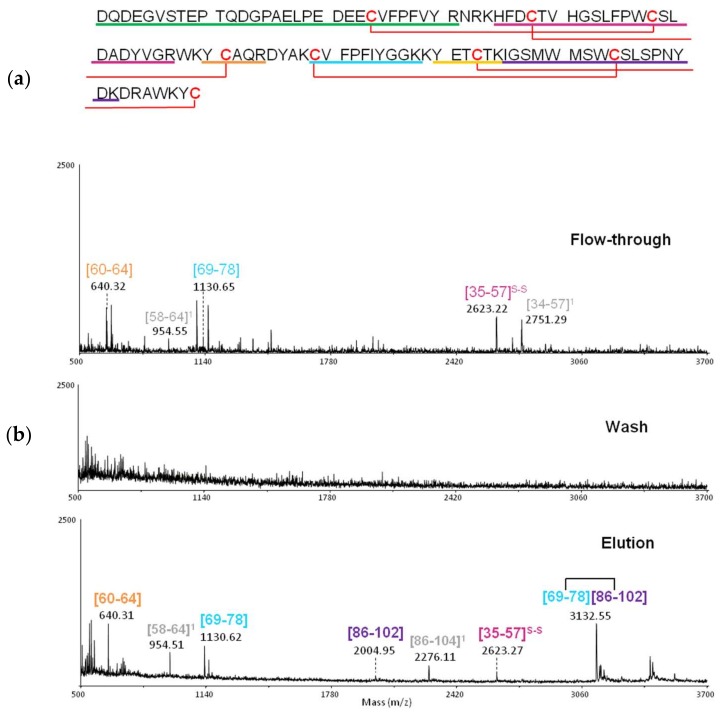
(**a**) PDC-109 sequence; peptide fragments obtained by trypsin digestion in solution are shown in colour; (**b**) MALDI-TOF MS spectra of the different fractions of the PDC-109 vs. Fuc-α1,4-GlcNAc CREDEX-MS excision experiment.

Once the functionality of the columns was confirmed, excision experiments with PDC-109 were run. Briefly, PDC-109 was incubated with the Fuc-α1,(3,4,6)-GlcNAc-DVS-Sepharose (individual columns) for 24 h, columns were washed until no protein was observed by MALDI-TOF MS, then lectin-sugar complexes were digested with trypsin at 37 °C overnight (Figure 7b, top panel; representative for Fucα-1,4-GlcNAc). Columns were again washed until no peptides were observed by MALDI-TOF MS (Figure 7b, middle panel), then the affinity-bound peptides were eluted with ACN:H_2_O (2:1 *v/v*, + 0.1% TFA) and identified by MALDI-TOF MS (Figure 7b, bottom panel). The mapped peptides are indicated in the primary structure of PDC-109 (Figure 7a).

In the Fuc-α1,4-GlcNAc excision experiment (Figure 7), five out of seven peptides (colored) identified in the elution fraction correlated directly with the predicted ones. In addition, two other peptides (highlighted in gray) observed in the elution fraction corresponded to miscleaved fragments. The only unique peptide in the excision experiments with all fucosylated glycoprobes was the disulfide-bridged fragment [69–78, 86–102], as well as its reduced components. Hence it is likely that these two peptides of the 2Fn2 domain, either disulfide-bound or individually, constitute the carbohydrate binding domain of PDC-109.

## 3. Discussion

Our SPR-based approach, using an extensive collection of well-defined glycopeptide probes exposing sugar epitopes in native-like form, has not only served to determine kinetic and affinity constants of these epitopes with specific plant lectins consistent with previously published results, but also shown its applicability to PDC-109, a fertilization-relevant protein in bovines, and possibly also humans. While the binding affinity of PDC-109 for heparin [32] or PC [26,33], as well as its chaperone-like activity [34] have received considerable attention, our analysis of the lectin-like activity of PDC-109 is, to the best of our knowledge, the first quantitative study of the carbohydrate binding behavior of PDC-109.

Although several glycoconjugates have been synthesized by other laboratories using also *N*[Me]-Aoa-peptide versions as mimics of natural glycoproteins, few of them have been used as probes for interaction studies [35,36], and not always with conclusive results. Thus, this work represents a further step to establish our SPR-approach as a reliable alternative in carbohydrate-lectin interaction studies. The complex nature of the natural PDC-109 preparation has exposed the difficulties in biomolecular interaction analysis when not dealing with well-defined and homogenous samples. Yet these challenges have also helped our understanding of biological phenomena where heterogeneous mixtures are common. The admitted limitations of the SPR approach in the particular case of PDC-109 have been satisfactorily overcome by CREDEX-MS, again showing both techniques as a suitable duet to decipher protein-carbohydrate interactions not only at the demonstration level [16] (i.e., with “academic” lectins) but with “real”, biologically relevant samples.

The PDC-109 lectin binding profile has been completed, and its specificity assessed against various validated glycosidic epitopes. Specifically, the interaction with common mammal epitopes has been qualitatively and quantitatively characterized by SPR, with a larger affinity found for the Fuc-α1,4-GlcNAc disaccharide, a result in agreement with previous studies which proposed that, during sperm reservoir formation, bovine sperm binds to an oligosaccharide ligand of the oviductal epithelium similar to the Lewis^a^ epitope (Galβ1-3[Fucα1-4]GlcNAc) [15,30]. On a different note, it is worth mentioning that unusual PDC-109 SPR sensorgrams posing difficulties for kinetic adjustment most likely reflected intrinsic PDC-109 properties, confirming that this protein is naturally a mixture of various isoforms and aggregation states that play an important role in modulating the interaction with other biomolecules [28,29,37,38,39]. In this context, self-aggregation of PDC-109 could be driven by the Neu5Ac-Hex-HexNAc trisaccharide found in roughly 50% of its isoforms in circulation. The lack of quantitative glycosylation might serve as a limiting factor in PDC-109-induced sperm coating, i.e., regulating the layer density around the spermatozoon.

The role of PDC-109 in the formation of the sperm reservoir, through its lectin specificity for carbohydrates containing Fuc, was corroborated by SPR and fine-tuned to Fuc-α1,(3,4,6)-GlcNAc by the orthogonal CREDEX-MS technique. In all experiments, a common peptide fragment has been identified that is very likely to be part of the PDC-109 CRD.

In conclusion, our study sheds new light on the interaction profile of the PDC-109 seminal plasma protein, and increases understanding of the molecular events involved in the capacitation and fecundation processes. Also importantly, such an understanding can make inroads into the design and development of novel fertility-related drugs.

## 4. Materials and Methods

### 4.1. Chemicals and Biological Samples

Fmoc (*N^α^*-(9-fluorenylmethyloxycarbonyl)) protected amino acids were purchased from Senn Chemichals (Dielsdorf, Switzerland). The dicyclohexylammonium (DCHA) salt of Boc (tertbutyloxycarbonyl)-methylaminooxyacetic acid was from NeoMPS (Strasbourg, France). Rink amide MBHA resin was from Novabiochem (Läufelfingen, Switzerland). 2-(1*H*-benzotriazol-1-yl)-1,1,3,3-tetramethyluronium hexafluorophosphate (HBTU) was obtained from Iris Biotech (Marktredwitz, Germany). *N*,*N*-diisopropylethylamine (DIEA) was from Merck (Darmstadt, Germany), and triisopropylsilane was from Sigma-Aldrich (Madrid, Spain). HPLC-grade acetonitrile (ACN), *N*,*N*-dimethylformamide (DMF), trifluoroacetic acid (TFA), diethyl ether, and pyridine were from SDS (Peypin, France). 

Carbohydrates (Gal-β1,(3,4,6)-GlcNAc, Man-α1,(2,3,6)-Man, Neu5Ac-α2,3-Gal-β1,4-GlcNAc, Neu5Ac-α2,6-Gal-β1,4-GlcNAc, Neu5Ac-α2,3/6-Gal-β1,4-Glc, Sialyl Lewis^x^ and Lewis^a^) employed in this work for glycopeptide synthesis were from Dextra (Reading, United Kingdom). Disaccharides with terminal Fuc (Fuc-α1,(3,4,6)-GlcNAc) were obtained from Toronto Research Chemicals (Toronto, Canada) and αMe-Fuc was from Iris Biotech GmbH (Marktredwitz, Germany).

Lectins from *Erythrina cristagalli* (ECA), *Maackia amurensis* (MAA), *Sambucus nigra* (SNA), *Canavalia ensiformis* (Con A), *Lotus tetragonolobus* (LTA) and *Ulex europaeus* (UEA) were purchased from Sigma-Aldrich (Madrid, Spain). Aniline, 2,5-dihydrobenzoic acid (DHB), and α-cyano-4-hydroxycinnamic acid (CHCA) were from Sigma-Aldrich (Madrid, Spain). Sinapinic acid (SA) was from Fluka (Madrid, Spain). Poros R2 was from Applied BioSystems (Foster City, CA, USA) and ZipTip^®^ Pipette Tips were from Merck Millipore (Merck KGaA, Darmstadt, Germany). Sequencing-grade modified porcine trypsin was from Promega (Madison, WI, USA). CM5 and C1 sensor chips, 1-ethyl-3-(3-diethylaminopropyl)-carbodiimide (EDC), *N*-hydroxysuccinimide (NHS), ethanolamine hydrochloride pH 8.5, and HBS-P (0.01 M HEPES pH 7.4; 0.15 M NaCl; 0.005% *v/v* surfactant P20) buffer were from Biacore (GE Healthcare, Uppsala, Sweden). Sepharose-4B, divinylsulfone and methyl-vinylsulfone were from Sigma-Aldrich (Madrid, Spain). Micro-columns and 35-µm pore size filters were from MoBiTec (Göttingen, Germany).

NuPAGE^®^ Novex^®^ 4–12% Bis-Tris precast polyacrylamide gels, NuPAGE^®^ MES and MOPS SDS running buffers, NuPAGE^®^ sample reducing agent and antioxidant, Colloidal Blue Staining Kit, BenchMark™ Protein Ladder, and Novex^®^ Sharp Unstained Protein Standard were from Invitrogen (Life Technologies, Carlsbad, CA, USA). Silver staining kit and Coomassie Brilliant Blue R-250 were from Bio-Rad Laboratories (Hercules, CA, USA).

PDC-109, purified from bull seminal plasma, was kindly donated by J.J. Calvete. Its purity was assessed by SDS-PAGE (15 and 18% acrylamide gels), where the protein appeared as two closely spaced bands of MW ~13 kDa, corresponding to the glycosylated and non-glycosylated forms. MALDI-TOF MS in the linear mode (SA matrix, positive polarity) also showed the two forms of PDC-109 and several oligomers. The concentration of purified PDC-109 was estimated from its extinction coefficient of 2.5 for a 1-mg/mL sample concentration at 280 nm [40].

N-deglycosylation of PDC-109 was done using PNGase F (1 µL; 250 units/0.25 mL) in phosphate buffer, 50 mM pH 7.3, 16 h, 37 °C. After filtration (10 kDa filter) to eliminate buffer salts, the efficiency of the procedure and the resulting compound/s was checked by SDS-PAGE and MALDI-TOF MS.

### 4.2. Peptide and Glycopeptide Synthesis

#### 4.2.1. Peptide Module Synthesis

The GFKKG-amide substrate was made by Fmoc solid-phase synthesis on a Rink amide MBHA resin (0.70 mmol/g) at a 0.1 mmol scale in an Applied Biosystems 430A automated synthesizer.

● *Boc-N[Me]-Aoa-OH coupling*

Prior to coupling to the GFKKG-amide resin, Boc-methylaminooxyacetic acid·DCHA salt (500 mg Boc-*N*[Me]-Aoa-OH/DCHA) was shaken with 0.1 M HCl and EtOAc (50 mL each) to release the free carboxyl form into the organic phase (3 extractions). After drying with MgSO_4_. EtOAc was evaporated and the residue used in manual coupling (3 equivalents each of Boc-amino acid and HBTU, 6 equivalents of DIEA) to the peptide-resin for 1 h, room temperature (r.t.), in DMF.

● *Peptide acetylation*

For SPR reference channel purposes, a fraction of the GFKKG-amide resin was acetylated with acetic anhydride (1 mmol; 94.5 μL) and DIEA (348.4 μL) in DMF for 1 h. 

● *Peptide cleavage and work-up*

Cleavage and deprotection of the peptide resins was done with TFA-water-triisopropylsilane (95:2.5:2.5, *v/v/v*, 90 min, r.t.). Peptides were isolated by precipitation with cold diethyl ether and centrifugation, then solubilized in water and lyophilized. The synthetic products (*N*[Me]-O-Aoa-GFKKG-amide and Ac-GFKKG-amide) were >95% pure by analytical RP-HPLC and had the correct mass by MALDI-TOF MS ([M+H]^+^_calc_~[M+H]^+^_exp_; 622.4 and 577.7 Da, respectively).

#### 4.2.2. Chemoselective Ligation of Carbohydrates

Conjugation between *N*[Me]-*O*-Aoa-GFKKG-amide peptide and oligosaccharides was done at 20 and 25 mM, respectively in 0.1 M NaOAc for 72 h at 37 °C and pH 3.5 for NAc-hexosamines or pH 4.6 for hexoses. Conjugations involving Fuc-containing disaccharides (Fuc-α1,(3,4,6)-GlcNAc) were performed both in the presence and the absence of 100 mM aniline as a nucleophile catalyst, to explore improvements in conjugation yields.

All glycoconjugates were purified by semi-preparative RP-HPLC on SphereClone C_18_ (Phenomenex, 250 mm × 10 mm; 5 μm) using a 10–20% linear gradient of acetonitrile into water (both eluents with 0.1% TFA). Immediately after purification, glycopeptide-containing fractions were neutralized with 10 mM NH_4_HCO_3_ (up to pH~5) to prevent acid degradation, and lyophylized. All synthetic di/trisaccharide-*N*[Me]-*O*-Aoa-GFKKG-amide glycopeptides were >90% pure by analytical RP-HPLC and had the expected mass by MALDI-TOF MS as shown Table 5.

### 4.3. High Performance Liquid Chromatography (HPLC)

RP-HPLC analysis and semi-preparative purifications were performed as follows:

Analytical separations were done in an LC-2010A system (Shimadzu, Kyoto, Japan) using Luna C_8_ (3 µm, 50 mm × 4.6 mm) and Sphereclone C_18_ (5 µm, 250 mm × 10 mm) columns (Phenomenex, Torrance, CA, USA) for peptides and glycopeptides, respectively. Linear gradients of solvent B (0.036% TFA in ACN) into solvent A (0.045% TFA in H_2_O) were used over 15 and 20 min, respectively, at a 1 mL/min flow rate.

Semi-preparative purifications were done in a SCL-10A system (Shimadzu) using Phenomenex Luna C_8_ (10 µm, 250 mm × 10 mm) and SphereClone C_18_ (10 µm, 250 mm × 10 mm) columns for peptides and glycopeptides, respectively. Linear gradients of solvent B (0.1% TFA in ACN) into A (0.1% TFA in H_2_O) over 30 and 20 min, respectively, were used, at a 5 mL/min flow rate.

### 4.4. MALDI-TOF Mass Spectrometry (MS)

Peptides and glycopeptides were dissolved in water and mixed with the corresponding matrix solution (1:1 *v/v*) and 1 µL of the mixture was applied to the MALDI target and allowed to dry at room temperature. For analysis of synthetic peptides and conjugates, DHB (10 mg/mL) in ACN:water:TFA (70:30:0.1 *v/v/v*) was chosen as matrix. For peptide digests, saturated CHCA in ACN:water:TFA (70:30:0.1 *v/v/v*) was used. For protein analysis, SA (10 mg/mL) in ACN:water:TFA (70:30:0.1 *v/v/v*) was used.

MS spectra were recorded on a Voyager-DE STR Biospectrometry workstation (Applied Biosystems, Foster City, CA, USA) equipped with a N_2_ laser (337 nm). Peptides and glycopeptides were measured in reflectron mode and positive polarity, except for Sia-containing probes, which were measured in both positive and negative modes. Proteins were measured in the linear mode and positive polarity. External calibration was performed using the Sequazyme^TM^ Peptide Mass Standard Kit (PerSeptive Biosystems, Framingham, MA, USA) of the desired range. Data were processed with the Data Explorer Software (Applied Biosystems).

### 4.5. Quantification Methods

#### 4.5.1. UV Quantification

Pure glycoprobes were quantified by UV-spectroscopy by measuring absorbance at 258 nm (λ_max_ of a Phe residue) using a Nanodrop device (Nanodrop Technologies, Inc., Wilmington, DE, USA). An operational extinction coefficient for the peptide *N*[Me]-*O*-Aoa-GFKKG-amide was experimentally obtained (ε 0.1438 mM^−1^ cm^−1^; l = 1 cm).

#### 4.5.2. Amino Acid Analysis (AAA)

As an alternative to UV quantification, AAA was used for both *N*[Me]-*O*-Aoa-GFKKG-amide and Ac-GFKKG-amide and glycopeptides (Table 5). Prior to AAA, ca. 10 nmol (10 μg) peptide or glycopeptide sample were dissolved in 200 μL of 12 M HCl and 200 μL of 0.1 M norleucine in water (20 nmol, internal standard) in a Pyrex glass tube. The tube was stoppered and hydrolysis was performed for 24 h at 110 °C. HCl was removed in a rotary evaporator prior to analysis of the hydrolysate by Gas Chromatography-Mass Spectrometry (Agilent Technologies, Barcelona, Spain). Amino acids (in both samples and calibration standards) were converted to tert-butyl dimethylsilyl (TBDMS) derivatives by treatment with 50 μL of *N*-tert-butyldimethylsilyl-*N*-methyltrifluoroacetamide (MTBSTFA) and 50 μL ACN for 4 h at 100 °C. Derivatized samples were next analyzed by GC-MS in an Agilent 6890N Network GC system fitted with an Agilent 5973N mass selective detector. A Phenomenex Zebron ZB-5 cross-linked 5% phenyl polymethyl siloxane capillary column (15 m × 0.25 mm i.d., 0.25 μm film thickness) was used. Oven temperature was initially held at 100 °C then raised to 310 °C at 25 °C/min. The interface and ion source temperatures were 150 and 230 °C, respectively. Column gas flow was 20 mL/min. The MS was operated in full scan mode (50–650 amu; scan rate 2.46 scans/s) with at least two characteristic mass fragments being recorded for each amino acid. Four replicates per sample were run. Quantification was performed from interpolation of calibration standard curves.

### 4.6. Surface Plasmon Resonance (SPR)

All SPR experiments were performed in a BIAcore 3000 instrument (GE Healthcare, Uppsala, Sweden) using carboxymethyl-functionalized CM5 or C1 sensor chips and HBS-P (0.01 M HEPES pH 7.4; 0.15 M NaCl; 0.005% *v/v* surfactant P20) as running buffer, supplemented when necessary with CaCl_2_ (5 mM) and MnCl_2_ (1 mM) or other competitive reagents/adjuvants such as bovine serum albumin (BSA), carboxymethyldextran (CMD) or polivinylpirrolidone (PVP). SPR sensorgram analysis and curve fitting were done with the BIAevaluation 4.1.1 sofware package (GE Healthcare, Uppsala, Sweden). In all experiments, specific binding responses were obtained by subtracting from each channel the reference channel response. Moreover, in most cases a double referencing was applied (i.e., reference Fc1 channel plus an internal reference standard, namely buffer injection subtraction).

#### 4.6.1. Immobilization of Glycoprobes

Glycoprobes immobilized on each sensor chip and their immobilization levels are given on Table 6. On each chip, a reference surface (preferentially, first flow cell) was created with a non-specific probe immobilized (*N*[Me]-*O*-Aoa-GFKKG-amide or Ac-GFKKG-amide peptides) to subtract the non-specific lectin binding to the dextran surface.

For all immobilizations, the carboxyl functionalities of the CM5 (or C1) sensor chip surfaces were activated by injecting a mixture of 0.05 M NHS and 0.2 M EDC (1:1 *v/v*; 50 µL) at 5 µL/min. Then, glycopeptide solutions were passed at 1–2 mg/mL in 10 mM NaOAc for approximately 12 min over the activated surface. For immobilization, pH 4 or pH 6 conditions were used, as required. In the specific case of PDC-109, protein solution was passed at 0.05 µg/µL in 10 mM NaOAc pH 4 over the activated surface. Afterwards, unreacted groups on all chip surfaces were blocked with ethanolamine-HCl (1 M; pH 8.5; 60 µL) or H_2_O (60 µL). The difference, in resonance units (RU), between readings after surface activation and final response was converted into amount of immobilized glycopeptide (0.981 RU~1 pg/mm^2^ for glycopeptides, 1 RU = 1 pg/mm^2^ for proteins; see legend Table 6).

#### 4.6.2. Binding and Kinetic Experiments

All kinetic measurements were carried out by running a manually programmed sequence of binding experiments using several protein range concentrations and two flow rates. Regeneration of the sensor surface was accomplished in a specific-manner by injecting the complementary carbohydrate (10 mM lac, 100–500 mM αMe-Man, 0.5 M αMe-Fuc or L-Fuc, 0.5 M GlcNAc, 0.5 M Glc, and 10 mM Sia). Alternatively, other procedures such as glycoprotein solutions (e.g., fetuin at 2–5 g/L), low pH-buffers (e.g., 10 mM glycine pH 1.5–2.5), high salt (e.g., 1 M NaCl), high pH or specific chemicals were also used to break the interaction.

For the evaluation of the sensor chips, binding experiments and kinetic studies were carried out with the various glycoprobes and specific well-characterized lectins (ECA, Con A, LTA, UEA-I, MAA, and SNA). Several concentrations of each lectin were prepared by serial 2/3-fold dilutions of the most concentrated sample in running buffer supplemented with CaCl_2_ (5 mM) and MnCl_2_ (1 mM). Thus, concentrations varied from 66 nM to 2.5 µM for ECA; from 2.5 to 80 nM for Con A; from 31.25 nM to 1 µM for LTA and UEA-I; from 250 nM to 1.9 µM for MAA and from 74 to 563 nM for SNA. Analyses were performed at 20 µL/min and kinetic studies at 10 and 40 μL/min. After lectin injection, the sample solution was replaced by running buffer and the dissociation phase was monitored for 3–6 min. Sensor surfaces were regenerated with a 10–50 µL-injection of 10 mM lac for ECA; 500 mM αMe-Man for Con A; 0.5 M L-Fuc for LTA and UEA-I and 5 g/L fetuin for MAA and SNA.

For determination of PDC-109 kinetic parameters, several concentrations in the 6.25–200 μΜ range were prepared by serial 2/3-fold dilutions of the most concentrated sample in HBS-P running buffer as a standard condition. Other buffer conditions (i.e., HBS-P buffer supplemented with CaCl_2_ (5 mM) and MnCl_2_ (1 mM), or adding other reagents such as BSA 0.5% carrier protein, CMD 0.5% and PVP 0.5% competitors) were tested to improve SPR sensorgrams and subsequent kinetic fittings. Binding experiments were performed at 25 °C and 30 μL/min flow rate. After PDC-109 injection (3-min pulse), the sample solution was replaced by running buffer and the dissociation phase was monitored for 6 min. Sensor surface was regenerated with a 60 μL-injection of specific carbohydrate (10 mM lac, 500 mM αMe-Man, 0.5 M αMe-Fuc, and 10 mM Sia) followed by a 60 μL-injection of NaCl 1 M.

For PDC-109 binding behavior studies, CM5 surface vs. C1 surface without dextran matrix were tested to assess the influence of this structural element. Binding experiments were performed by injecting both PDC-109 untreated and after deglycosylation at 50 µM, 25 °C, and 20 μL/min flow. After protein injection, the sample solution was replaced by running buffer and the dissociation phase was monitored for 4 min. Sensor surfaces were regenerated with a 30 μL-injection of 1 M NaCl. Alternatively, PDC-109 aggregation tests were monitored by SPR. Briefly, protein solution of 50 µM PDC-109 was passed over immobilized PDC-109 and reference flow cells at 20 μL/min. After dissociation phase, surface regeneration was accomplished by injection of 30 μL of 1 M NaCl. Two replicates were performed for each injection in all binding behavior studies.

### 4.7. CREDEX-MS

Prior the CREDEX-MS experiment, UEA-I (20 µg) and PDC-109 (20 µg) were digested in solution with trypsin and the following incubation conditions were used: 1 µg trypsin in 25 mM NH_4_HCO_3_, pH 8.5 (1:20 enzyme:substrate ratio), overnight at 37 °C for UEA-I. Otherwise, 0.4 µg trypsin in 25 mM NH_4_HCO_3_, pH 8.5 (1:50 enzyme:substrate ratio), overnight at 37 °C for PDC-109. The resulting peptide mixtures were desalted by reverse phase micro-column (Poros R2) or by ZipTip^®^ Pipette Tips, and analyzed by MALDI-TOF MS.

For disaccharide immobilization, 5 mg of sugar (Fuc-α1,(3,4,6)-GlcNAc for UEA-I/PDC-109) was dissolved in 50 µL of 0.5 M K_2_CO_3_ (pH 11), and the solution was incubated in a microcolumn with 50 µg of dry DVS-activated Sepharose, overnight at r.t. under continuous shaking (800 rpm). Then, the microcolumn was washed sequentially with 50 mM NH_4_OAc (pH 4) and 50 mM NH_4_HCO_3_ (pH 8). Finally, it was equilibrated with binding buffer (10 mM HEPES, 25 mM NaCl, 5 mM CaCl_2_ and 1 mM MnCl_2_) and stored at 4 °C.

Before performing excision experiments, columns were tested for correct binding. Briefly, 20 µg of UEA-I or PDC-109 were added onto the Fuc-α1,(3,4,6)-GlcNAc-Sepharose columns, and were incubated in binding buffer for 24 h at 37 °C. Unbound materials were removed by extensive washing with running buffer. Bound lectins were eluted with 60% ACN (0.1% TFA), and the protein content of each fractions (flow through; wash and elution) was analyzed by 1D-SDS-PAGE electrophoresis.

For excision experiments, 20 µg of UEA-I or PDC-109 in running buffer (75 μL) were loaded onto the Fuc-α1,4-GlcNAc-Sepharose microcolumn and incubated for 24 h at 37 °C. Unbound lectin was removed by washing with running buffer until no signal could be detected by MS. Then, the sugar-lectin complex was digested overnight with trypsin (enzyme:substrate ratios as above) in 25 mM NH_4_HCO_3_, pH 8.5 at 37 °C. After 15 h, the flow through containing digestion products was removed and the column was washed. After washes with running buffer, specific-bound peptides were eluted with 60 µL of 60% ACN (0.1% TFA), concentrated and lyophilized. Prior to MALDI-TOF analysis, each sample was desalted by means of micro-column purification (Poros R2) or by ZipTip^®^ Pipette Tips.

## Figures and Tables

**Table 1:** Carbohydrate epitopes commonly encountered in mammalian specimens and corresponding lectins with well-defined binding properties.

Epitope	Carbohydrate	Lectin
Disaccharides with terminal β-galactose	Gal-β1,4-Glc	*Erythrina cristagalli* (ECA)
	Gal-β1,4-GlcNAc	
	Gal-β1,3-GlcNAc	
	Gal-β1,6-GlcNAc	
Mannobioses	Man-α1,2-Man	*Concanavalin A* (Con A)
	Man-α1,3-Man	
	Man-α1,6-Man	
Fucosylated disaccharides	Fuc-α1,3-GlcNAc	*Lotus tetragonolobus* (LTA) and *Ulex europeaus I* (UEA I)
	Fuc-α1,4-GlcNAc Fuc-α1,6-GlcNAc	
Neu5Ac-containing oligosaccharides	Neu5Ac-α2,3-Gal-β1,4-GlcNAc	*Maackia amurensis* (MAA)
	Neu5Ac-α2,6-Gal-β1,4-GlcNAc	*Sambucus nigra* (SNA)
	Neu5Ac-α2,3/6-Gal-β1,4-Glc	MAA and SNA

Abbreviations for monosaccharides: Gal; galactose, Glc; glucose, GlcNAc; *N*-acetyl glucosamine, Man; mannose, Fuc; fucose, Neu5Ac; 5-*N*-acetyl neuraminic acid.

**Table 2:** Kinetic constants of the interaction of Con A with mannobiose-containing glycoprobes determined by SPR.

Ligand	k_a1_ (M^−1^ s^−1^)	k_d1_ (s^−1^)	k_a2_ (RU^−1^ s^−1^)	k_d2_ (s^−1^)	K_A1_ (M^−1^)
Man-α1,2-Man-Aoa(m)-GFKKG	1.68 × 10^4^	1.33 × 10^−1^	2.88 × 10^−2^	7.06 × 10^−2^	1.26 × 10^5^
Man-α1,3-Man-Aoa(m)-GFKKG	2.28 × 10^4^	6.61 × 10^−2^	3.78 × 10^−3^	6.29 × 10^−2^	3.45 × 10^5^
Man-α1,6-Man-Aoa(m)-GFKKG	8.91 × 10^3^	2.89 × 10^−2^	7.15 × 10^−4^	1.90 × 10^−2^	3.08 × 10^5^

**Table 3:** Kinetic constants of MAA and SNA to their specific sialic acid-containing glycoprobes determined by SPR.

Lectin	Ligand	k_a_ (M^−^^1^ s^−^^1^)	k_d_ (s^−^^1^)	K_A_ (M^−^^1^)
MAA	Neu5Ac-α2,3-lacNAc	5.55 × 10^3^	6.08 × 10^−^^3^	9.12 × 10^5^
SNA	Neu5Ac-α2,6-lacNAc	1.02 × 10^4^	1.63 × 10^−^^3^	6.27 × 10^6^

**Table 4:** Kinetic rate constants and derived association (K_A_) constant of PDC-109 to various neo-glycoprobes as determined by SPR.

Ligand Immobilized	k_a_ (M^−1^ s^−1^)	k_d_ (s^−1^)	K_A_ (M^−1^)
Gal-β1,4-GlcNAc-Aoa(m)-GFKKG	8.62 × 10^1^	8.60 × 10^−3^	1.00 × 10^4^
Gal-β1,3-GlcNAc-Aoa(m)-GFKKG	1.28 × 10^2^	7.56 × 10^−3^	1.70 × 10^4^
Gal-β1,6-GlcNAc-Aoa(m)-GFKKG	4.72 × 10^2^	2.08 × 10^−2^	2.27 × 10^4^
Man-α1,2-Man-Aoa(m)-GFKKG	1.02 × 10^3^	1.30 × 10^−2^	7.87 × 10^4^
Man-α1,3-Man-Aoa(m)-GFKKG	3.86 × 10^2^	4.40 × 10^−3^	8.77 × 10^4^
Man-α1,6-Man-Aoa(m)-GFKKG	4.10 × 10^2^	1.73 × 10^−2^	2.38 × 10^4^
Fuc-α1,3-GlcNAc-Aoa(m)-GFKKG	7.63 × 10^2^	1.65 × 10^−3^	2.37 × 10^5^
Fuc-α1,4-GlcNAc-Aoa(m)-GFKKG	2.91 × 10^3^	8.83 × 10^−5^	4.93 × 10^6^
Fuc-α1,6-GlcNAc-Aoa(m)-GFKKG	6.45 × 10^2^	3.45 × 10^−3^	7.31 × 10^4^
Neu5Ac-α2,3-Gal-β1,4-GlcNAc-Aoa(m)-GFKKG	9.99 × 10^1^	4.77 × 10^−3^	1.24 × 10^4^
Neu5Ac-α2,6-Gal-β1,4-GlcNAc-Aoa(m)-GFKKG	1.88 × 10^3^	2.53 × 10^−3^	1.66 × 10^5^
Neu5Ac-α2,3/6-Gal-β1,4-Glc-Aoa(m)-GFKKG	2.12 × 10^3^	8.10 × 10^−3^	2.77 × 10^4^

**Table 5:** Synthetic neo-glycoprobes displaying different oligosaccharides.

Glycopeptide	Conversion * (%)	t_R_^#^ (min)	[M+H]^+^ calc	[M+H]^+^ exp
Gal-β1,4-GlcNAc-Aoa(m)-GFKKG	69	7.07	987.50	987.86
Gal-β1,3-GlcNAc-Aoa(m)-GFKKG	51	7.34	987.50	987.94
Gal-β1,6-GlcNAc-Aoa(m)-GFKKG	33	6.58	987.50	987.85
Man-α1,2-Man-Aoa(m)-GFKKG	12	6.72	946.48	946.41
Man-α1,3-Man-Aoa(m)-GFKKG	42	6.56	946.48	946.39
Man-α1,6-Man-Aoa(m)-GFKKG	43	7.23	946.48	946.62
Fuc-α1,3-GlcNAc-Aoa(m)-GFKKG	9	7.79	971.51	971.35
Fuc-α1,4-GlcNAc-Aoa(m)-GFKKG	16	7.80	971.51	971.36
Fuc-α1,6-GlcNAc-Aoa(m)-GFKKG	55	8.30	971.51	971.58
Neu5Ac-α2,3-Gal-β1,4-GlcNAc-Aoa(m)-GFKKG	54	6.26	1279.60	1279.48
Neu5Ac-α2,6-Gal-β1,4-GlcNAc-Aoa(m)-GFKKG	46	6.16	1279.60	1279.63
Neu5Ac-α2,3/6-Gal-β1,4-Glc-Aoa(m)-GFKKG	47	5.54	1223.55	1223.79

* Estimated by integration of HPLC peak areas at 220 nm; ^#^ Retention times in analytical RP-HPLC with linear gradients from 10 to 20% solvent B into A over 20 min (B: 0.036% TFA in ACN; A: 0.045% TFA in H_2_O), at flow rate of 1 mL/min.

**Table 6:** Sensor chips prepared during this work.

Sensor Chip	Fc Channel	Ligand Immobilized	Amount Immobilized	
			RU	pmol/mm^2 (#)^
1	1	Ac-GFKKG	73	0.13
	2	Galβ1,4GlcNAc-Aoa(m)-GFKKG	28	0.03
	3	Galβ1,3GlcNAc-Aoa(m)-GFKKG	149	0.15
	4	Galβ1,6GlcNAc-Aoa(m)-GFKKG	191	0.20
2	1	Ac-GFKKG	20	0.04
	2	Manα1,2Man-Aoa(m)-GFKKG	300	0.32
	3	Manα1,3Man-Aoa(m)-GFKKG	805	0.87
	4	Manα1,6Man-Aoa(m)-GFKKG	718	0.77
3	1	Ac-GFKKG	195	0.34
	2	Fucα1,3GlcNAc-Aoa(m)-GFKKG	81	0.09
	3	Fucα1,4GlcNAc-Aoa(m)-GFKKG	31	0.03
	4	Fucα1,6GlcNAc-Aoa(m)-GFKKG	296	0.31
4	1	Ac-GFKKG	178	0.31
	2	Siaα2,3lacNAc-Aoa(m)-GFKKG	323	0.26
	3	Siaα2,6lacNAc-Aoa(m)-GFKKG	444	0.35
	4	Siaα2,3/6lac-Aoa(m)-GFKKG	91	0.08
5 ^(^*^)^	1	Ac-GFKKG	-	-
	2	EDC/NHS–EtNH_2_^(‡)^	-	-
6	1	EDC/NHS–EtNH_2_^(‡)^	-	-
	2	PDC-109	1645	0.09

Sia = Neu5Ac; lac = Gal-β1,4-Glc; lacNAc = Gal-β1,4-GlcNAc; ^(#)^ We questioned if the manufacturer’s suggested equivalence of 1 RU = 1 pg of immobilized analyte, devised for globular proteins, was indeed also applicable to our present case, where the ligands are short glycopeptides. To clarify this point, specific refractive index for the peptide was calculated from amino acid compositions by the Lorentz–Lorenz equation [41,42]. Thus, 0.981 RU = 1 pg equivalences were calculated for GFKKG; ^(^*^)^ C1 chip (unique): provides the same functionality as sensor chips CM5 (the rest of used chips) but has no dextran matrix (the carboxyl groups are attached directly to the surface layer); ^(‡)^ A simple activation and deactivation surface control.

## Abbreviations

BSP	bovine seminal plasma protein
Con A	*Concanavalin A* (*Canavalia ensiformis)*
CRD	carbohydrate recognition domain
CREDEX	Carbohydrate REcognition Domain EXcision
CV	coefficient of variation
DVS	divinylsulfone
ECA	*Erythrina cristagalli* agglutinin
Fuc	Fucose
Hex	Hexose
HPLC	high performance liquid chromatography
ITC	isothermal titration calorimetry
LTA	*Lotus tetragonolobus* agglutinin
MAA	*Maackia amurensis* agglutinin
MALDI-TOF	matrix assisted laser desorption ionization time of flight
MS	mass spectrometry
PC	phosphatidylcholine
RU	resonance units
SDS-PAGE	sodium dodecyl sulfate polyacrylamide gel electrophoresis
Sia	sialic acid (N-acetylneuraminic acid, Neu5Ac)??
SNA	*Sambucus nigra* agglutinin
SPR	surface plasmon resonance
UEA-I	*Ulex europaeus* agglutinin I
